# *Chlamydia psittaci* pneumonia in a patient using ustekinumab therapy for Crohn’s disease

**DOI:** 10.1128/asmcr.00129-25

**Published:** 2026-01-09

**Authors:** J. A. M. C. Dirks, L. Mulder, K. Burgers, J. G. M. C. Damoiseaux, E. R. Heddema

**Affiliations:** 1 Department of Medical Microbiology (and reference laboratory for human Chlamydia infections from animal origin), Zuyderland Medical Center 159205, Sittard-Geleen, the Netherlands; 2 Pulmonology Department, Zuyderland Medical Center 159205, Heerlen, the Netherlands; 3 Department of Sexual Health, Infectious Diseases and Environmental Health, Public Health Service South Limburg 26146 , Heerlen, the Netherlands; 4 Central Diagnostic Laboratory, Maastricht University Medical Center, Maastricht, the Netherlands; Rush University Medical Center, Chicago, Illinois, USA

**Keywords:** opportunistic infection, biologic therapy, *Chlamydia psittaci*, ustekinumab

## Abstract

**Background:**

Ustekinumab, a monoclonal antibody targeting the p40 subunit of interleukin-12 and interleukin-23, is widely used for immune-mediated diseases such as Crohn’s disease. By modulating Th1 and Th17 pathways, ustekinumab may increase susceptibility to intracellular pathogens. We report the first case of *Chlamydia psittaci* pneumonia in a patient treated with ustekinumab.

**Case Summary:**

A 27-year-old woman with Crohn’s disease presented with severe community-acquired pneumonia, progressive hypoxemia, and pulmonary embolism 3 weeks after initiating ustekinumab therapy. Despite broad-spectrum antibiotics, clinical improvement was limited. Her exposure history revealed contact with multiple pet birds, several of which died recently. Polymerase chain reaction testing confirmed *C. psittaci* genotype A infection. Targeted treatment with doxycycline led to clinical recovery. Ustekinumab therapy was temporarily postponed.

**Conclusion:**

This case highlights a potential association between ustekinumab and susceptibility to *C. psittaci*, a zoonotic intracellular pathogen. It underscores the importance of exposure history and considering atypical pathogens in immunocompromised patients. Clinicians should maintain vigilance for opportunistic infections in individuals receiving targeted biologic therapies.

## INTRODUCTION

Ustekinumab is a monoclonal antibody that targets the p40 subunit shared by interleukin-12 (IL-12) and interleukin-23 (IL-23), which are key cytokines involved in the differentiation and maintenance of Th1 and Th17 cells, respectively. By inhibiting the activity of these cytokines, ustekinumab selectively suppresses key immune pathways involved in several immune-mediated diseases and can be used to treat moderate to severe plaque psoriasis, psoriatic arthritis, ulcerative colitis, and Crohn’s disease. The Th1 and Th17 pathways are crucial for the immune response to intracellular pathogens, as has been shown in individuals genetically deficient for IL-12/IL-23 cytokine pathways (1, 2). Consequently, case reports have associated ustekinumab use with intracellular pathogens like *Mycobacterium tuberculosis* and *Legionella pneumophila* (3, 4). This is the first case report describing an infection with *Chlamydia psittaci*, a strict intracellular pathogen, in a patient treated with ustekinumab.

## CASE PRESENTATION

**Fig 1:**
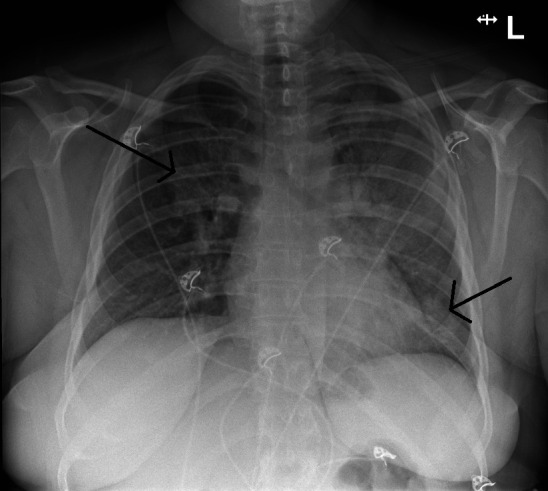
Posterior-anterior chest X-ray made during presentation at the emergency department showing consolidations in the left lobe and in the right upper lobe (indicated by arrows).

**Fig 2:**
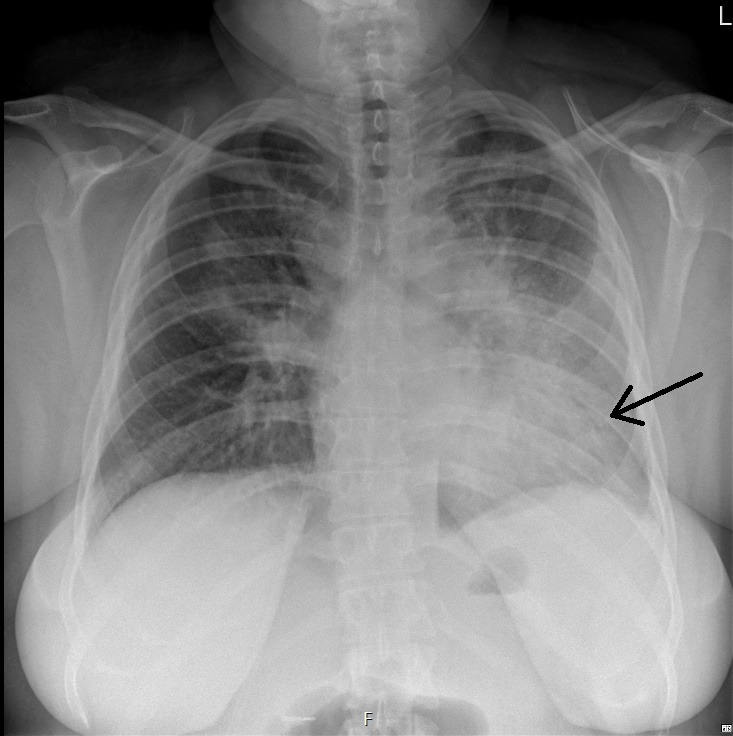
Posterior-anterior chest X-ray made on day 5 of admission showing consolidations in the left lobe (indicated by the arrow).

A 27-year-old woman was referred to the emergency department by her general practitioner because of progressive dyspnea. The patient had a history of Crohn’s disease and started with standard ustekinumab treatment (520 mg i.v.) 3 weeks ago, which made her immunocompromised. She presented with complaints of high fever (>40°C), dyspnea, dry cough, arthralgia, myalgia, and a loss of appetite for 5 days. Physical examination upon admission revealed no rash, a body temperature of 39.4°C, pulse rate of 130 bpm, blood pressure of 129/89 mmHg, and vesicular breath sounds in both lungs, and she required 3 L/min of supplemental oxygen to reach an oxygen saturation of 96%. Arterial blood gas analysis showed respiratory alkalosis with hypoxemia (8.4 kPa). Laboratory findings included an elevated CRP of 414 mg/L (reference [ref] <10), leukocytosis (13.1 × 10⁹/L, ref. 4.5–11.0), hyponatremia (130 mmol/L, ref. 135–145), elevated ASAT (203 U/L, ref. <31) and ALAT (67 U/L, ref. <34), and acute kidney injury (creatinine 93 µmol/L, ref. 48–91; eGFR 73, ref. >90). Chest X-ray showed consolidations in the left lobe and in the right upper lobe (Fig. 1). The patient was diagnosed with community-acquired pneumonia, admitted to the pulmonology department, and started on ceftriaxone (2 g i.v. once daily) and ciprofloxacin (500 mg PO twice daily). A chest CT scan showed consolidation foci in both lungs, primarily in the left lung . Pneumococcal and *L. pneumophila* serogroup I urinary antigen tests were negative. On day 5 of admission, the patient still required oxygen supplementation; CRP stayed elevated (133 mg/L); and the patient showed only slow clinical improvement. A bronchoscopy was considered but not performed due to the potential risks of respiratory failure. On day 8 of admission, due to the lasting hypoxemia, diagnostic testing for a pulmonary embolism was done. A CT pulmonary angiography showed pulmonary embolisms. Treatment with rivaroxaban 15 mg PO twice daily was started. A further history revealed that the patient took care of several parakeets, cockatoos, and parrots, one of which recently died due to an unidentified illness. Due to the X-ray configuration (Fig. 2) and the clinical history of the patient, there was the suspicion of *C. psittaci* infection. On day 5 of admission, additional diagnostic testing was done by PCR for *C. psittaci* on a throat swab (eSwab). Because the consolidations in both lungs were decreasing, laboratory results showed a decreasing CRP (39 mg/L), normalization of the leukocyte count (7.5 × 10⁹/L), and improvement in kidney function, antibiotic treatment was stopped after 7 days. In the afternoon of day 8 of admission, *C. psittaci* was detected by PCR. As ciprofloxacin in a dose of 500 mg bid does not provide adequate *C. psittaci* coverage due to the relatively high MIC (5), doxycycline was started (loading dose 200 mg, then 100 mg bid) for six more days. During admission, the gastroenterologist was consulted about whether or not to continue ustekinumab. The advice was to continue it when the patient had clinically and biochemically improved, defined as declining CRP, normalization of leukocyte count, resolution of hypoxemia, and radiographic improvement. The decision was made to postpone the following subcutaneous injection by 2 weeks. The patient recovered clinically and was discharged from the hospital. After discharge, sequencing part of the outer-membrane protein showed that the infection was caused by *C. psittaci* genotype A.

## DISCUSSION

This report describes a rare case of a *C. psittaci* infection in a patient undergoing treatment with ustekinumab, raising pertinent clinical and immunological considerations regarding the risk of opportunistic infections in patients receiving targeted biologic therapy (6). Although causality cannot be definitively inferred, the presentation raises concern that ustekinumab therapy may predispose patients to infections with intracellular pathogens. The possible association between ustekinumab and infection with an obligate intracellular pathogen such as *C. psittaci* warrants particular attention in the context of community-acquired pneumonia.

*C. psittaci* is an obligate intracellular bacterium known for causing psittacosis, a zoonotic infection that can be transmitted to humans, predominantly from birds, particularly parrots and pigeons. The severity of the disease can range from none or mild flu-like symptoms to life-threatening disease (pneumonia with respiratory failure, pericarditis, and fulminant sepsis). The incubation period of *C. psittaci* infection is usually 5–14 days but can be up to 1 month (7). With adequate antibiotic treatment, mortality is less than 1% (7).

*C. psittaci* was estimated to cause approximately 1%–4% of hospitalized cases with community-acquired pneumonia, often with a recent history of bird exposure (8, 9). However, the precise incidence of psittacosis is difficult to establish, likely due to lack of routine testing, scarcity of commercially available nucleic acid amplification tests, available testing facilities, unfamiliarity with the disease, and undiagnosed cases in primary care (10). The diagnosis of psittacosis can be established with serologic tests, PCR, and even metagenomics on respiratory samples. While samples such as sputum or bronchoalveolar lavage fluid are preferred over oropharyngeal swab, these can be positive, as was the case in our patient, but false-negative results are common (11, 12). Culture facilities handling *C. psittaci* are scarce and not routinely available.

Historically, first recognized by serotyping, *C. psittaci* can be classified into various genotypes, each with a more or less preference for a specific host. There are currently 10 recognized *C. psittaci* genotypes (A-F, E/B, WC, M56, Mat116) based on the gene sequence coding for the outer-membrane protein (*ompA*, MOMP) (13). In the Netherlands, genotypes A (parrots) and B (pigeons) are most frequently found in reported patients with a *C. psittaci* infection (14). If indicated, for example, in an outbreak setting, additional genotypic analyses can be performed, such as multilocus sequence typing (15).

Several aspects are noteworthy in this case. The patient was only 27 years old, much younger than the average psittacosis patient in the Netherlands (median age 67 [IQR 58–74]) (16). Leukocytosis was present, while WBC counts in psittacosis are often normal (17, 18). Furthermore, our patient developed pulmonary embolisms, which are rarely described in *C. psittaci* infections (19). It is thought to occur because of the host inflammatory response, rather than a direct effect of *Chlamydia* itself; damage to endothelial cells triggers inflammation and activates the coagulation cascade, facilitating the formation of thrombi (20).

Ustekinumab is generally associated with a low incidence of serious infections (21–24). The relatively low incidence of serious infections with ustekinumab likely reflects its selective inhibition of IL-12/IL-23 pathways, in contrast to broader immunosuppressive agents like cyclophosphamide or corticosteroids. Comparison with other monoclonal antibodies that target distinct molecules in the type 1 cytokine pathway, relevant for the clearance of intracellular pathogens, may be hampered by differences in blocking proximal versus distal molecules in the pathway. Nevertheless, by neutralizing the shared p40 subunit of IL-12 and IL-23 and thereby impairing IL-12 and IL-23 function, ustekinumab may attenuate both innate and adaptive immune responses. IL-12 promotes differentiation of naïve CD4^+^ T cells into Th1 cells, which produce interferon-gamma (IFN-γ), a key activator of macrophages required for killing intracellular pathogens. IL-23 supports Th17 cell maintenance, which contributes to mucosal defense. Specifically, the decrease in IFN-γ impairs macrophage activation and intracellular bacterial clearance, thereby increasing susceptibility to intracellular pathogens (4), as has been reported for *M. tuberculosis* and *L. pneumophila* (3, 4, 6). There is almost no published data on other types of immunosuppression and *C. psittaci* infections (25–27). Thus, our case of *C. psittaci* infection in a patient on ustekinumab adds to the sparse literature on psittacosis in the context of contemporary immunosuppressive therapy.

*C. psittaci* is primarily transmitted through inhalation of aerosolized secretions such as feather dust or fecal material from infected birds. Investigation by the regional Municipal Health Service identified multiple pet birds housed in an indoor aviary at the patient’s home, including two lovebirds (*Agapornis* spp.), two barred parakeets (*Bolborhynchus lineola*), four cockatiels (*Nymphicus hollandicus*), and three Kakariki (*Cyanoramphus* spp.). Two of these Kakariki birds had been acquired a few weeks prior to the onset of illness in the birds’ owner, of which one became ill around the same time as our patient and subsequently died. Samples taken by the Dutch Food and Consumer Product Safety authority confirmed the presence of *C. psittaci* genotype A in several of the birds, following PCR testing. In accordance with Dutch regulations and recommendations, all birds were euthanized to prevent further zoonotic transmission. It is unknown if the new Kakarikis were sick when they were purchased, but it is plausible to assume the new birds introduced this pathogen with subsequent spread to the other birds and finally our patient. Importantly, the absence of known bird exposure does not exclude the diagnosis, as such exposure can go unnoticed. During the 2023–2024 winter, European countries reported an increase in *C. psittaci* infections, without obvious bird exposure or contact with bird excreta (28).

This case emphasizes the need for heightened awareness of intracellular pathogens such as zoonotic *C. psittaci* infections in patients receiving biologic therapies like ustekinumab. While its targeted mechanism results in a relatively lower overall infection risk compared to traditional immunosuppressants, clinicians should recognize proneness to intracellular pathogens. A detailed exposure history should be routinely obtained prior to initiation of biologic therapy; however, diagnostic vigilance is warranted in patients even without known risk factors or apparent bird contact.
